# Effect of Mucilage Extracted from *Corchorus olitorius* Leaves on Bovine Serum Albumin (BSA)-Stabilized Oil-in-Water Emulsions

**DOI:** 10.3390/polym15010113

**Published:** 2022-12-27

**Authors:** Do-Yeong Kim, Hyunsu Kim

**Affiliations:** Department of Food Science and Biotechnology, Dongguk University-Seoul, 32, Dongguk-ro, Ilsandong-gu, Goyang-si 10326, Gyeonggi-do, Republic of Korea

**Keywords:** *Corchorus olitorius* L., mucilage, bovine serum albumin, O/W emulsion, emulsifying stability

## Abstract

The present study examined the effect of mucilage extracted from *Corchorus olitorius* L. leaves on the emulsifying stability of bovine serum albumin (BSA)-stabilized oil-in-water (O/W) emulsions during the storage for seven days. O/W emulsions were prepared with a 90% aqueous phase containing *C. olitorius* mucilage (0–1.00% *w*/*v*) together with 0.5% (*w*/*v*) BSA and 10% oil phase. Emulsion properties were analyzed by measuring droplet size, zeta potential, spectroturbidity, backscattering profiles (%BS), and visual observations. The mean droplet size of emulsions prepared with 0.75 and 1.00% mucilage did not show significant changes during storage. The zeta potential of all the emulsions exhibited a negative charge of approximately −40 mV, but electrical repulsion was not the dominant stabilization mechanism in the emulsion. *C. olitorius* mucilage was able to increase the viscosity of the aqueous phase of the O/W emulsion system, which prevented droplet flocculation and enhanced the emulsion stability against phase separation at higher concentrations. The most stable emulsions during the storage period were those with 1.00% *C. olitorius* mucilage. In conclusion, *C. olitorius* mucilage has good potential for the preparation of stable O/W emulsions and can be used as a plant-based natural emulsifying and thickening agent in the food industry.

## 1. Introduction

Emulsions are widely used in various fields, such as food, cosmetics, and pharmaceuticals. Generally, oil-in-water (O/W) emulsions are composed of a discontinuous oil phase dispersed into a continuous water phase. Emulsions are thermodynamically unstable systems and prone to phase separation with physical phenomena such as flocculation, coalescence, and creaming [1,2]. In order to delay these destabilization processes, emulsifying or stabilizing agents can be used. Emulsifying agents are surface active materials that adsorb between the oil–water interface and promote the formation of small oil droplets in the O/W emulsion, thereby lowering the interfacial tension during homogenization [3].

Recently, natural biopolymers such as proteins and polysaccharides have been used to replace synthetic emulsifiers because of their biodegradability and non-toxicity [4]. Bovine serum albumin (BSA) is frequently used as an emulsifier in food emulsions because of its amphiphilic nature and flexible conformation. BSA consists of three homologous domains (I, II, and III) with different pH-dependent net charges that can affect how the protein adsorbs to the surface of oil droplets [5]. Moreover, BSA plays an essential role in food emulsions as a multiphasic system compared to protein alone. Previous studies have shown that combining BSA with charged polysaccharides, by forming BSA-polysaccharide complexes can help stabilize emulsions, e.g., BSA/sugar beet pectin, BSA/fucoidan, and BSA/chondroitin sulfate [6,7,8,9].

*Corchorus olitorius* L. is a green-yellow plant that is widely grown in tropical Asia and Africa [10], and its leaves are commonly used for nutritional and medicinal purposes in India [11,12]. Several studies have reported that the leaves have diverse biological activities such as antitumor [13], antimicrobial [14,15], and antioxidant activity [16] due to the presence of high amounts of β-carotene, ascorbic acid, lutein, and phenolic compounds such as quercetin [17]. In addition, *C. olitorius* leaves contain a large amount of mucilage, which has unique functional properties and bioactive roles [18,19].

Mucilage is a water-soluble biopolymer that consists of various neutral sugars and uronic. Quantitatively, the predominant component in the *C. olitorius* L. mucilage was uronic acids, followed by rhamnose, galactose, arabinose, glucose, xylose, and fucose [18]. However, various factors can influence the monosaccharide composition of mucilage, such as the part of the plant (seed or leaf), cultivation environment conditions, extraction conditions, etc. Currently, plant-derived mucilages have been reviewed with much attention to their characteristics, functional attributes, and bioactive roles [20]. Plant-based mucilages are valuable natural polysaccharides with a wide range of applications as thickeners, emulsion stabilizers, suspending, gelling, and film-forming agents in the food and pharmaceutical industries [20,21,22,23,24]. Mucilage can be extracted from different plant parts, and its physicochemical and functional properties are highly dependent on the extraction conditions, plant material, and cultivation environment [20]. There are various reports on the potential use of plant mucilage as a new alternative source for application in food emulsions, such as mucilages extracted from *Pereskia aculeata* Miller leaves [3], *Guazuma ulmifolia* Lam. seeds [25], *Salvia hispanica* L. seeds [26], *Abelmoschus esculentus* L. pods [27], and so on. However, there is very little information on the use of mucilage extracted from *C. olitorius* L. leaves, especially on its effect when added to emulsions. In our previous study, emulsions prepared with mucilage extracted from *C. olitorius* leaves exhibited a higher emulsion stability index at higher concentrations, although lower than that of xanthan gum. However, there is a limitation in using mucilage alone as an emulsifying agent for long-term emulsion stabilization because mucilage is a hydrophilic polymer with a high molecular weight and low surface activity [18]. Therefore, in this study, BSA is used as an emulsifying agent in the preparation of emulsion. In addition, two steps: both homogenization and ultrasonication, were used to prepare the emulsions. The objective of this study was to investigate the effect of adding different concentrations of *C. olitorius* mucilage on the stability of O/W emulsions formulated with BSA as a function of the storage period.

## 2. Materials and Methods

### 2.1. Materials

*Corchorus olitorius* L. leaves powder, which was ground after drying the leaves without any treatment, was purchased from Dusonae Yackcho (Seoul, Korea). BSA (#A4503), iodine, potassium iodide (KI), ruthenium red, and lead (II) acetate trihydrate were purchased from Sigma-Aldrich (St. Louis, MO, USA). 1-Naphthol, sodium phosphate dibasic anhydrous, sodium dihydrogen phosphate anhydrous, sodium azide, ethanol, and concentrated sulfuric acid (H_2_SO_4_) were purchased from Samchun Chemical Co., Ltd. (Gyeonggi-do, Korea). Corn oil, used as the oil phase, was purchased from a local supermarket. Distilled water was obtained from a Milli-Q water purification system (Billerica, MA, USA). All chemical reagents were of analytical grade.

### 2.2. Extraction of Mucilage

**Figure 1:**
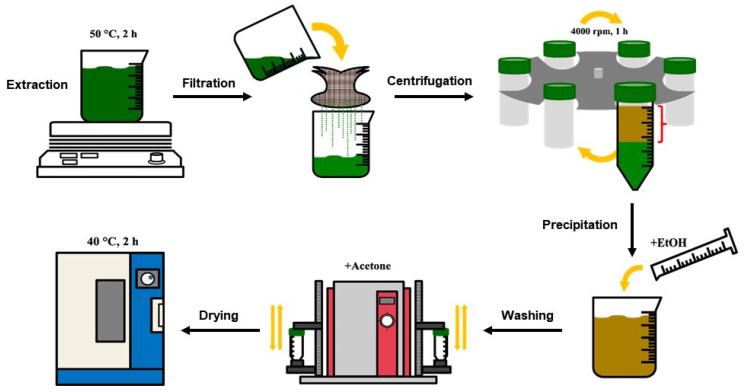
Extraction process of *C. olitorius* mucilage.

Mucilage was extracted from *C. olitorius* leaves as described in our previous work [18], and the schematic of the extraction process is shown in Figure 1. *C. olitorius* leaf powder (10 g) was soaked in distilled water (200 mL) with stirring at 50 °C for 2 h. The dissolved solution was separated using a muslin cloth and centrifuged (MF80, Hanil Science Ind., Seoul, Korea) at 4000 rpm for 1 h. The supernatant was precipitated using five volumes of ethyl alcohol, and the precipitated mucilage was washed using two volumes of acetone for removing chlorophyll by repeated shaking and centrifuging (2700 rpm, 15 min). Afterward, the precipitated mucilage was dried in an oven (SW-90D, Sang Woo Scientific Co., Gyeonggi-do, Korea) at 40 °C for 2 h and ground using a blender. The powdered *C. olitorius* mucilage was sealed in a tube and stored in desiccators for use in the emulsion experiment. According to our previous research [18], the yield and molecular weight of *C. olitorius* mucilage were 10.52% and 1.9 × 10^6^ Da, respectively. The monosaccharide composition showed that rhamnose (23.8%) and uronic acid (34.3%) were major components, and other monosaccharides such as galactose (14.0%), arabinose (10.8%), glucose (7.8%), xylose (7.3%), and fucose (2.1%) were also contained in the mucilage.

### 2.3. Confirmation of Mucilage Presence

The extracted *C. olitorius* mucilage was subjected to confirmatory tests according to the method previously described in [28]. In order to determine the presence of carbohydrates (Molisch’s test), 4 mL of *C. olitorius* mucilage solution (0.25% *w*/*v*) was placed in a test tube, and then five drops of Molisch’s regent (a solution of 1-naphthol in 95% ethanol) was added. The solution was then slowly poured into a tube containing 1 mL of concentrated sulfuric acid and left for 2 min. For the iodine test to determine the absence of starch, *C. olitorius* mucilage was mixed with 1 mL of 0.2 N iodine solution, and color change was observed. For the ruthenium test, a small quantity of mucilage powder was taken and mounted on a slide with two drops of ruthenium red solution (ruthenium red in 10% lead acetate solution). Afterward, it was observed under an inverted microscope (Olympus IX70, Tokyo, Japan).

### 2.4. Preparation of Oil-in-Water Emulsion

Emulsions were prepared according to the method described by Kim and Shin [6] with slight modifications. The BSA stock solution was prepared by dissolving BSA powder in a buffer solution (10 mM sodium phosphate buffer, pH 7.4). Mucilage dispersions were prepared by dissolving mucilage powder into distilled water under stirring at 300 rpm for 12 h to ensure complete hydration. Oil-in-water (O/W) emulsions were prepared by mixing 90% of the aqueous phase (0.5% BSA, 10 mM sodium phosphate buffer, mucilage concentrations of 0.25, 0.50, 0.75, and 1.00% *w*/*v*) and 10% of the corn oil using a homogenizer (HG-15D, Daihan Scientific, Seoul, Korea) at 15,000 rpm for 3 min. Then, the coarse samples were sonicated at 20 kHz for 2 min by using a probe-type ultrasound processor (VC750, Sonics & Materials Inc., Newtown, CT, USA) to produce finer emulsions. In order to prevent temperature effects, the tubes containing the emulsion samples were placed in an ice bath during sonication. Emulsion with BSA alone was prepared as a control. Sodium azide (0.01% *w*/*v*) was added to the emulsions to avoid microbial growth in storage experiments.

### 2.5. Characterization of Emulsions

#### 2.5.1. Droplet Size and Zeta Potential of Emulsions

The z-average droplet size and zeta potential of the emulsion samples were measured using a Zetasizer (Nano-ZS90, Malvern Instruments Ltd., Worcestershire, UK). Before the analysis, 20 μL of the emulsion was taken from the bottom of the sample tube and diluted to a ratio of 1:300 (*v*/*v*) in buffer solution (10 mM phosphate buffer, pH 7.4) to avoid multiple scattering effects. All samples were analyzed immediately after preparation and during storage for seven days in triplicate.

#### 2.5.2. Spectroturbidity of Emulsions

The turbidity of the emulsion samples was measured using a spectrophotometer (UV-1800, Shimadzu, Kyoto, Japan) at 500 nm for seven days. Before analysis, the emulsion sample was taken from the bottom of the tube and diluted approximately 500-fold using 10 mM phosphate buffer. Distilled water was used as a blank reference, and all samples were run in triplicate.

#### 2.5.3. Creaming Stability of Emulsions

The physical stability of the emulsion samples was determined using a TurbiScan Lab (Formulaction, Toulouse, France), according to Kim and Shin [6,7]. Emulsion samples were transferred to a cylindrical glass tube immediately after preparation and monitored for 3 h with a scan every 5 min at 25 °C. The creaming stability of each sample was represented as backscattering (%BS) profiles as a function of time and the Turbiscan stability index (TSI). TSI, a Turbiscan-specific parameter, was calculated as the sum of all of the destabilization processes occurring in the studied emulsion and indicated how fast the droplets contained in a colloidal dispersion sample sediment. Therefore, the lower the TSI value at a specific time point, the more stable the sample can be considered, and it was used to compare and characterize the physical stability of the emulsion samples [29,30].

### 2.6. Statistical Analysis

All tests were carried out in triplicate, and the data were statistically analyzed using SPSS Statistics 27 (SPSS, Inc., Chicago, IL, USA). Significant differences between mean values were determined by one-way analysis of variance (ANOVA) followed by Duncan’s multiple range test at a significance level of *p* < 0.05.

## 3. Results and Discussion

### 3.1. Conformation of Mucilage Extracted from Corchorus olitorius *L.*

**Figure 2:**
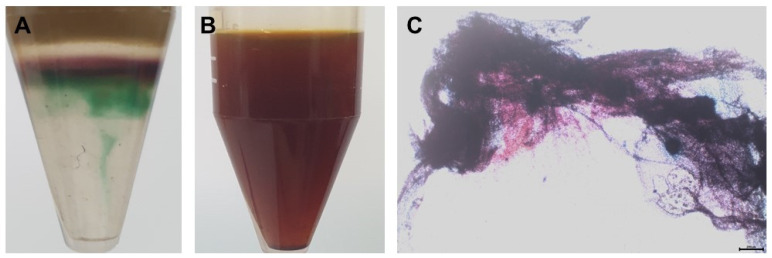
Confirmation of *C. olitorius* mucilage: (**A**) Molisch’s test, (**B**) iodine test, and (**C**) ruthenium red test.

The results of the confirmatory tests of the extracted *C. olitorius* mucilage are presented in Figure 2. In these tests, the mucilage reacts with Molisch’s reagent to form a purple-colored ring at the junction of the two liquids, indicating the presence of carbohydrates (Figure 2A). No color change was observed in the iodine test, indicating the presence of polysaccharides and the absence of starch (Figure 2B). When the extracted powder was treated with a solution of ruthenium red, it showed a red color confirming the presence of mucilage (Figure 2C).

### 3.2. Droplet Size of Emulsions

Table 1 shows the mean droplet size of dispersed oil droplets in emulsions prepared with different concentrations of *C. olitorius* mucilage (0–1.00%) for seven days. The average droplet sizes of the emulsions studied in this work ranged from 266 nm to 659 nm. This could be attributed to the sonication treatment characteristics used, which allow for obtaining small droplet sizes at the nanoscale [31,32]. In the case of an emulsion formulation with Pereskia aculeate Miller mucilage, droplet sizes from 116 nm to 385 nm were also reported when using sonication techniques [3].

For fresh emulsion, the mean droplet size of the emulsion prepared with only BSA was measured to be 297 nm, and the size increased significantly (*p* < 0.05) with increasing mucilage concentration up to 0.25%. This fact may be related to the flocculation of droplets in the protein-polysaccharide stabilized emulsion through a depletion mechanism at a low mucilage concentration [1]. Furthermore, the size of the droplets remained constant with increasing concentrations of *C. olitorius* mucilage (0.50–1.00%). Notably, the high viscosity of the aqueous phase produced by the *C. olitorius* mucilage affected the homogenization process and prevented the complete disruption or distribution of the oil droplets. Similar results were observed in previous studies [3,33].

**Figure 3:**
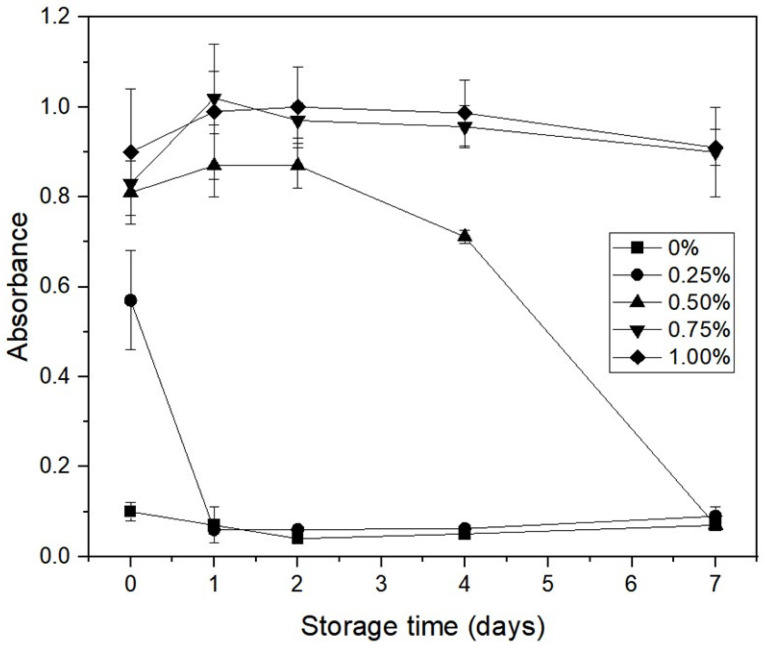
Spectroturbidity of O/W emulsions containing different concentrations of *C. olitorius* mucilage during seven days of storage.

**Figure 4:**
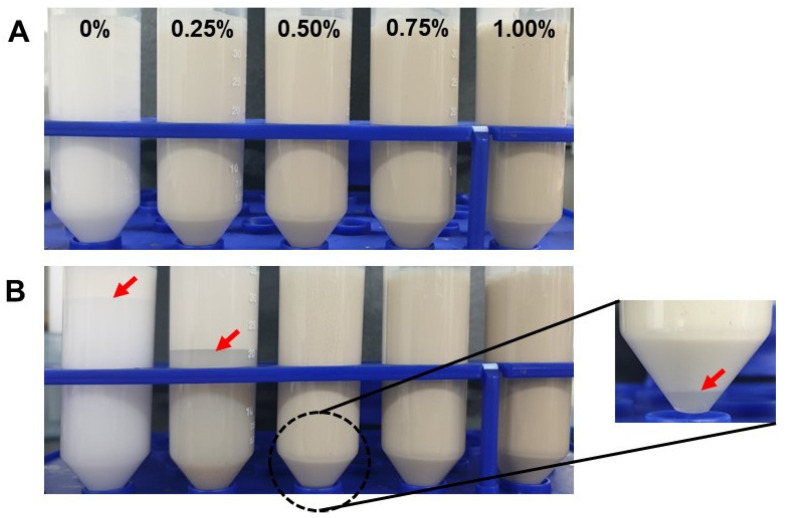
Visual observation of O/W emulsions containing different concentrations of *C. olitorius* mucilage: (**A**) fresh emulsion and (**B**) emulsions after seven days of storage.

At a storage time of seven days, the formation of a larger droplet population was observed in the emulsion formulated with BSA alone, reaching a maximum size of 448 nm. The droplet size of the emulsions formulated with 0.25% and 0.50% *C. olitorius* mucilage showed a significant decrease with storage time, especially on days 1 and 4, respectively. This is because the emulsion sample was taken from the bottom of the tube for droplet size measurement, and at that time, phase separation of the emulsion had already occurred, which seems to be a reflected result. This was consistent with the turbidity results and visual observation (Figure 3 and Figure 4, respectively) of emulsions with a low mucilage concentration, where the turbidity decreased at the bottom of the tube. In addition, the disruption of oil flocs during emulsion dilution for droplet size analysis could be the reason for not observing droplet size growth [34]. In contrast, the average droplet size of emulsions prepared with 0.75 and 1.00% mucilage did not show significant changes during storage. This could be associated with the effect of the increased viscosity of the continuous phase of the emulsion system due to the high mucilage concentration, which prevents the mobility and collision frequency of the oil droplets and thus maintains their integrity. These findings are consistent with the previous study. Capitani et al. [35] reported that the droplet size of sodium caseinate-stabilized emulsions with a high mucilage concentration (0.8%) remained constant throughout the storage time.

### 3.3. Zeta Potential of Emulsions

The zeta potential values (mV) of the BSA-stabilized emulsions with mucilage (0–1.00%) as a function of storage time are presented in Table 2. For all the storage periods studied, the mean zeta potential values of the emulsions with and without mucilage were negatively charged, as both BSA and mucilage are negatively charged above the isoelectric point of BSA (pI = 4.6–4.8). BSA has a net charge of −18 with the domains having individual charges of −10, −8, and 0, respectively, at neutral pH [36] and *C. olitorius* mucilage showed a negative charge regardless of concentration and pH according to our previous study [18]. For the emulsion immediately after preparation, the electrical charge of BSA-coated oil droplets at pH 7.4 was −41.43 mV, and it became slightly negative as the mucilage concentration of the aqueous phase of the emulsion increased.

In this study, the zeta potential values of the emulsions stabilized only with BSA were significantly reduced after seven days, suggesting that the molecular structure of BSA was rearranged at the interface and, at the same time, the charge integrity was altered at the interface [6]. Interestingly, the zeta potential of emulsions with added *C. olitorius* mucilage remained almost unchanged during storage time. This may be due to the charge screening effect that occurs between the two biopolymers. This finding is in agreement with the results reported by [33] for the stabilized emulsion of whey protein concentrate in the presence of gum extracted from *Lepidium perfoliatum* seeds. Although no differences with respect to storage time were observed in the zeta potential value of the emulsions, phase separation occurred in the emulsion to which a low concentration of mucilage was added, and no phase separation occurred in the emulsion to which a high concentration (≥0.75%) of mucilage was added (Figure 4). In the latter case, it is possible that, although both biopolymers are negatively charged, some of the positive charges of BSA initially interact with the anionically charged mucilage and form a strong electrostatic repulsion between the oil droplets, which is then saturated with an excessive negative charge sufficient to prevent droplet flocculation, resulting in excellent stability during storage [37]. However, considering the results obtained in this study, it was clear that the dominant stabilization mechanism of the *C. olitorius* mucilage in the emulsion was not electrical repulsion.

### 3.4. Spectroturbidity of Emulsions

Figure 3 shows the changes in turbidity of emulsions containing different concentrations of *C. olitorius* mucilage and 0.5% BSA during seven days of storage. Emulsions without *C. olitorius* mucilage showed very low turbidity during storage. Considering the visual observation (Figure 4), creaming in the emulsions without mucilage appeared to proceed rapidly on day 1, and the boundary line gradually became clearer until approximately day 7. The turbidity of the emulsion with a low mucilage concentration (<0.75%) decreased during storage time. In particular, the turbidity decreased remarkably on days 1 and 4 for the emulsion containing 0.25% and 0.50% mucilage, respectively, which is in agreement with those obtained by visual observation (Figure 4). Emulsions prepared with 0.25% mucilage showed an opaque layer with a boundary height of more than half of the sample tube after seven days of storage. Phase separation was also observed in emulsions stabilized with 0.50% mucilage after seven days of storage, presenting a reduction in turbidity in the lower part of the sample tube (Figure 4). The destabilization that occurred at low mucilage concentration could be due to depletion flocculation. Similarly to this phenomenon, the presence of low gum concentration has been shown to improve the rate of droplet coalescence and emulsion creaming due to depletion flocculation [38].

Conversely, emulsion turbidity maintained a plateau at high mucilage concentrations (≥0.75%) during storage without phase separation (Figure 4). A similar observation was also reported in a study involving chia mucilage-stabilized O/W emulsions [39]. They found that the addition of 0.75% chia mucilage to the emulsions contributed to greater emulsion stability against creaming over 120 days. They attributed that the formation of a three-dimensional network by chia mucilage could help decrease the mobility of oil droplets. According to previous studies, one of the advantages of other emulsion stabilization mechanisms using biopolymers is their ability to increase the viscosity of the aqueous phase to prevent coalescence, thereby reducing oil droplet mobility [40,41]. In our previous study, the viscosity of *C. olitorius* mucilage increased with increasing concentration and 1% mucilage solution showed approximately 150 cP, which was similar to xanthan gum (XG) at 0.2% (*w*/*v*) [18]. According to previous research, the emulsion prepared with XG was completely stable with no creaming or phase separations in the presence of 0.12 and 0.2% XG due to the formation of an oil droplet network as well as modification of the viscosity of the continuous phase [42]. In this sense, the emulsion with *C. olitorius* mucilage had greater viscosity than BSA alone and, therefore, the higher emulsion stability observed in this study could have resulted from a gradual increase in viscosity as the mucilage content was increased from 0.25 to 1.00% in the aqueous phase of the emulsion system. In addition, there was a higher proportion of high molecular weight mucilage molecules in the aqueous phase, which favored the formation of three-dimensional structures, suppressing the mobility and collision frequency of droplets, thus increasing flow resistance and preventing creaming [43]. In this study, creaming stability was additionally measured without diluting the emulsion using Turbiscan to confirm the difference between the two samples at high concentrations.

### 3.5. Creaming Stability of Emulsions

**Figure 5:**
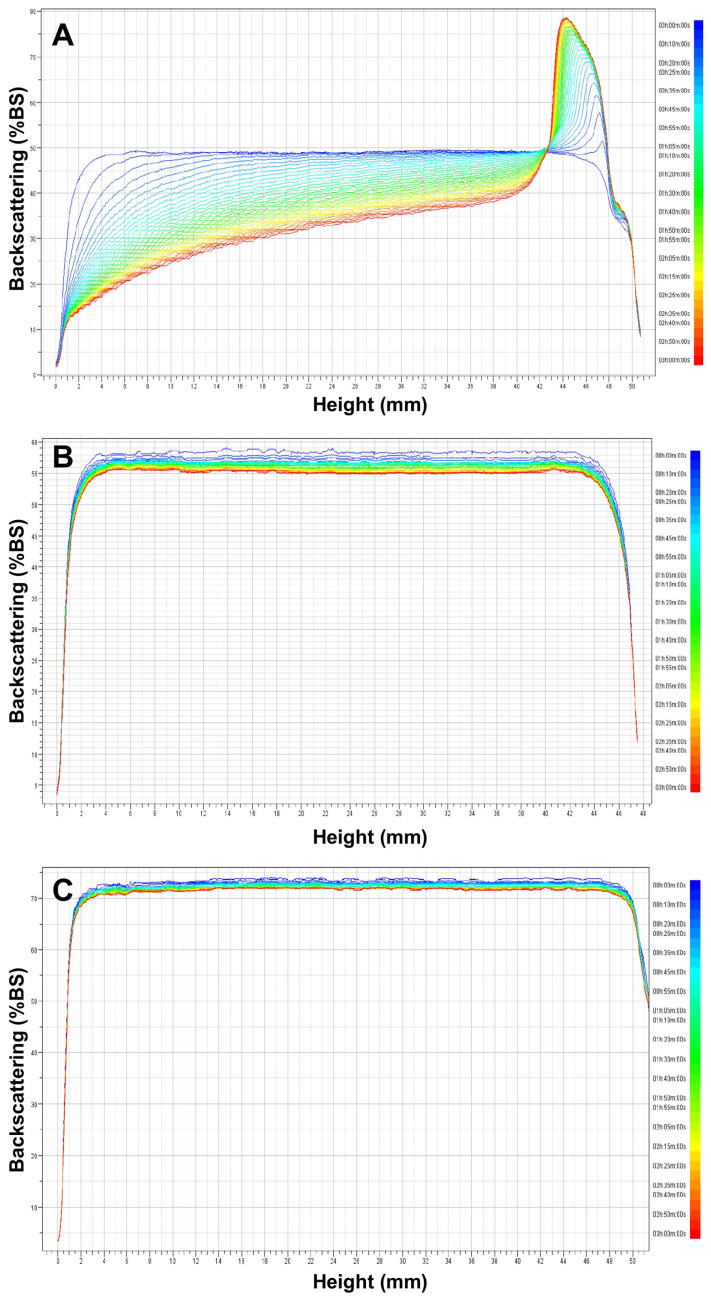
Backscattering profiles versus sample height (mm) of O/W emulsions with the addition of *C. olitorius* mucilage: (**A**) 0%, (**B**) 0.75%, and (**C**) 1.00%. The different colored lines represent the scanning curve in different times.

**Figure 6:**
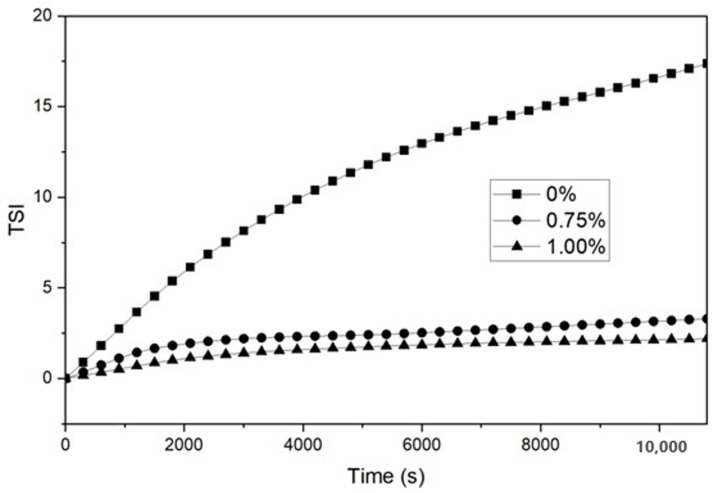
Turbiscan stability index (TSI) of O/W emulsions with the addition of *C. olitorius* mucilage for 3 h of storage at room temperature.

Figure 5 shows the backscattering (%BS) profiles obtained for O/W emulsions with and without the addition of 0.75 and 1.00% *C. olitorius* mucilage. The %BS signals back to the direction from which they came, indicating that the emulsion was destabilized by creaming, as the signal increased in the upper part of the sample cell (cream layer) and decreased in the lower part of the cell (clarification) [44]. As can be seen in Figure 5A, the emulsion without mucilage shows a shift in the BS profile at the top and bottom of the tube during the measurement time, indicating that the %BS decreased due to the lower droplet concentration at the bottom of the tube. Conversely, it shows that creaming occurred because the %BS increased towards the top of the tube. In contrast, the profiles shown in Figure 5B,C remained constant, without changes in relation to tube height or storage time. Similar behavior was observed in O/W emulsions formulated with 0.75% chia mucilage [26]. The high stability of the emulsions with a higher mucilage concentration (0.75 and 1.00%) can be attributed to the increased viscosity of the continuous phase. We also evaluated the effectiveness of *C. olitorius* mucilage as an emulsifying agent using a Turbiscan stability index (TSI), as shown in Figure 6. The emulsion without mucilage had a TSI value of 17.4 after 3 h, which was higher than that of the emulsion with mucilage. Conversely, the emulsions containing 0.75% and 1.00% mucilage showed the same trend according to the storage period but with TSI values of 3.3 and 2.2, respectively, with a slight difference in the exact stability of each sample. Therefore, the data clearly showed that the emulsion stabilized with 1.00% *C. olitorius* mucilage was slightly more stable than the emulsion with 0.75% mucilage.

## 4. Conclusions

The droplet size of emulsions prepared with *C. olitorius* mucilage was larger with increasing mucilage concentration, and the addition of *C. olitorius* mucilage had no significant effect on zeta potential values during storage time. This study showed that the addition of a low concentration of *C. olitorius* mucilage (≤0.50%) to BSA-stabilized O/W emulsions at neutral pH caused flocculation and coalescence of the oil droplets, leading to phase separation. However, the addition of *C. olitorius* mucilage at concentrations ≥0.75% improved the emulsifying stability of O/W emulsions during storage time. Based on the %BS profile obtained, the emulsion stabilized with 1.00% *C. olitorius* mucilage and presented remarkable stability during storage, having a TSI value of 2.2. This suggests that an increase in mucilage concentration is associated with an increase in solution viscosity and could contribute positively to emulsion stability due to a reduction in oil droplet coalescence. However, further studies on the flow behavior of emulsions with *C. olitorius* mucilage are needed to accurately confirm the dominant stabilization mechanism. Currently, most of the available research has focused on the characterization of *C. olitorius* mucilage. As the mucilage extracted from the leaves of *C. olitorius* is not yet well utilized, the result of this study provides practical information on the functionality of novel hydrocolloids that exhibit potent emulsifying stability. Additionally, it can be concluded that *C. olitorius* mucilage could be used as an alternative emulsifying agent in the cosmetic, pharmaceutical, and food industries.

## Figures and Tables

**Table 1:** Droplet size (nm) of O/W emulsions containing different concentrations of *C. olitorius* mucilage during seven days of storage.

Mucilage (%)	Storage Time (Days)				
	0	1	2	4	7
0	297.00 ± 15.7 0 ^bC^	336.63 ± 87.40 ^bB^	273.56 ± 22.34 ^bC^	333.57 ± 15.00 ^bC^	448.03 ± 5.36 ^aB^
0.25	538.37 ± 55.18 ^aB^	266.80 ± 3.87 ^cB^	277.77 ± 16.40 ^cC^	347.77 ± 20.90 ^bC^	295.90 ± 44.49 ^bcD^
0.50	607.10 ± 60.75 ^aAB^	597.60 ± 36.25 ^aA^	657.77 ± 17.42 ^aA^	497.77 ± 24.16 ^bB^	368.37 ± 38.10 ^cC^
0.75	580.33 ± 22.25 ^abAB^	603.57 ± 33.19 ^abA^	633.73 ± 18.49 ^aAB^	567.07 ± 39.64 ^bA^	552.60 ± 22.87 ^bA^
1.00	659.73 ± 68.69 ^aA^	626.60 ± 19.24 ^abA^	615.03 ± 31.28 ^abB^	591.70 ± 23.62 ^abA^	573.33 ± 30.13 ^bA^

Results are shown as mean ± standard deviation (n = 3). a–c Values followed by different lowercase letters differ significantly (*p* < 0.05) as a function of storage time (days). A–D Values followed by different capital letters differ significantly (*p* < 0.05) as a function of mucilage concentration (%).

**Table 2:** Zeta potential (mV) of O/W emulsions containing different concentrations of *C. olitorius* mucilage during seven days of storage.

Mucilage (%)	Storage Time (Days)				
	0	1	2	4	7
0	−41.43 ± 0.35 ^bA^	−40.26 ± 0.23 ^bA^	−37.33 ± 1.35 ^aA^	−42.47 ± 1.97 ^bA^	−48.80 ± 2.48 ^cB^
0.25	−44.87 ± 1.03 ^bB^	−44.77 ± 0.32 ^bB^	−45.50 ± 0.92 ^bB^	−42.17 ± 1.47 ^aA^	−43.83 ± 1.47 ^abA^
0.50	−44.33 ± 2.40 ^abB^	−44.87 ± 0.61 ^bB^	−45.27 ± 0.96 ^bB^	−41.80 ± 1.55 ^aA^	−42.80 ± 0.78 ^abA^
0.75	−44.23 ± 0.21 ^abB^	−44.80 ± 1.40 ^abB^	−46.40 ± 2.01 ^bB^	−42.43 ± 0.97 ^aA^	−43.47 ± 1.86 ^aA^
1.00	−43.13 ± 0.80 ^aAB^	−44.80 ± 0.75 ^abB^	−46.13 ± 0.38 ^bB^	−43.50 ± 1.15 ^aA^	−43.83 ± 1.60 ^aA^

Results are shown as mean ± standard deviation (n = 3). a–c Values followed by different lowercase letters differ significantly (*p* < 0.05) as a function of storage time (days). A–B Values followed by different capital letters differ significantly (*p* < 0.05) as a function of mucilage concentration (%).

## Data Availability

The data presented in this study are available in the article.
